# Practical Notes

**Published:** 1877-05-20

**Authors:** 


### Practical Notes.

QUININE PILLS.—Our co-laborer, Dr. Dyer, writes against the use of quinine in pills, particularly those put up for sale. He remarks: One of our physicians was attending a patient near this place, and gave a quinine pill every two hours. After two days, the fever being worse, it was found that the pills passed the bowels without being dissolved. A solution of quinine was then used with success. He also writes of

PUEPERAL HEMORRHAGICA.

May 12th was called to see Mrs. D.,—multiparo—infant one year old. Found her bleeding from nose, gums, mouth, and bowels. Is menstruating very slightly, the first time since birth of child. Signs of puerperal hemorrhagica; stigmata scattered all over her body: petechial, vibices and echymometa. Has scurvey. Look upon the case as one of vicarious menstruation. R. Fl. ext. ergot i dr. every two hours; also powders of sugar of lead and Doveri to alternate; the juice of two lemons per day.

13th no better; 14th hemorrhage slacking: third day of her menstrual flow gave hot baths to increase the flow, but no medicine internally, for fear of hemorrhage that I might not control.

15th much better. To continue treatment with 5 gr. doses of soluble citrate of iron and quinine every four hours during day. Will give iron until next menstrual period.

Dec. 7th Mrs. D. was a few weeks confined all right.

WINE BITTERS.—Pure Claret wine at meals is used very extensively in France and is said to be promotive of digestion. Combined with nux vomica, as in the annexed recipe, it constitutes an excellent tonic and appetizer:—

R. Fl. ext. Cinchonæ......dr. i.
Tinc. nucis vom........gtt. v.
Syrup aurantii cort....oziss.
Claret wine..............oz. viii.

M. Take two to four tablespoonsful before eating.

SPERMATORRHŒA.—Prof. Hayes, of Philadelphia, says he knows no better treatment for spermatorrhœa, than phosphorus and strychnia.

R. Strichniæ Sulphas....gr. ij;
Phosphori......... .....gr. j.

To make fifty pills. One three times daily.

The diet to be nutritious, but not rich; the suppers light; the bladder kept well emptied, and the rectum free from irritation. (Naphey.)

The same treatment answers well for impotence. When spermatorrhœa can be traced to irritable prostate astringent injections are advised.

R. Zinc. acetatis.........gr. iv;
Water...... ............oz. iv. M.

Use twice daily, and on going to bed.

Prof. Gross, of Philadelphia, prescribes the following for spermatorrhœa:

R. Elix. cinchonæ.........dr. ss.
Acedi nitrici duliti...gtt. viij.
Strychniæ sulph......gr. 1 16

This quantity to be taken three times per day. The dose of strychnia seems full large.

NEURALGIC PILL.—

R. Quiniæ sulphatis......gr. i.
Ferri et potassæ tart..gr. ij.
Morphiæ sulph..gr. 1-24 ad 1-12

M.—Take every hour until an expected paroxysm has been missed.

Recommended by Mr. Gregory and Dr. Burdenell Carter, in periodic neuralgia, l. c., p. 121.—*New Remedies.*

DIABETES.—In Naphey's late work on Therepeutics, we find that carbonate of ammonia is mentioned with great favor ln the treatment of diabetes:—

R. Ammonia carbonatis dr.ij to iv.
Aqua cinnamomi.....oz.iv.

M. Take tablespoontul three or four times a day, with a moderately restricted diet. Also,

R. Tinct. ferri chloride......gttxx.
Well diluted with water thrice daily.

In this disease Dr. La Costa, relies mainly on ½ gr. doses of opium repeated three times per day.

CHRONIC SORE THROAT.—Prof. Pancoast, uses as a favorite gargle in chronic sore throat, the following formula:

R. Tincturæ myrrhae.....

Tinc. krameriae........

Mellis despumatae..aa oz. j.

Acidi muriat. diluti.. gtt. xv.

ANOTHER.

R. Acidi tanici............dr. ss.

Mellis rosae...........oz. jss.

Aquae.....................oz. ivss.

FOLICULAR PHARYNGITIS.—There is a form of sore throat, often obstinate,which when examined will be found to be attended with numerous folicular pimples in the posterior wall of the pharynx, for this Prof. Da Costa, of Philadelphia, uses the following:

R. Sulphate of copper....dr. j.
Water....................oz. j. M.

Apply with a brush three times a week.

ANTI-EMETIC.—As an excellent anti-emetic, particularly in uterine affections use:

R. Tinc. nux vomica.....dr. i.

Tinc. Ginger...........dr. ij.

DOSE.—Gtt xv in a little mint water every half hour until four or five doses are taken.

